# TeleZK-FL: enabling trustless and verifiable remote patient monitoring via quantized zero-knowledge federated learning

**DOI:** 10.3389/fdgth.2026.1821129

**Published:** 2026-08-05

**Authors:** Prabhavathi Jayaraman, Radhakrishnan Delhibabu

**Affiliations:** School of Computer Science and Engineering, Vellore Institute of Technology, Vellore, Tamil Nadu, India

**Keywords:** data privacy, edge computing, federated learning, medical AI, quantization, remote patient monitoring, telehealth, zero-knowledge proofs

## Abstract

**Background:**

The rapid expansion of Internet of Medical Things (IoMT) and telehealth platforms has generated vast amounts of patient data suitable for training diagnostic Artificial Intelligence (AI) models. However, strict privacy regulations (HIPAA, GDPR) and the risk of data breaches prevent the centralization of this sensitive information. While Federated Learning (FL) allows for collaborative training without sharing raw patient data, it introduces a critical “trust deficit”: central aggregators cannot verify the integrity of local model updates without inspecting the private data, leaving the system vulnerable to model poisoning and malicious actors.

**Methods:**

We introduce *TeleZK-FL*, a privacy-preserving framework designed specifically for resource-constrained telehealth environments. Unlike existing Zero-Knowledge Proof (ZKP) systems that require high-performance computing or offload proof generation to trusted edge servers, TeleZK-FL integrates (1) Post-Training Quantization (PTQ) to compress model gradients from 32-bit floating-point to 8-bit integers, and (2) optimized Look-Up Table (LUT) arguments to generate cryptographic proofs of client-side training integrity directly on edge devices. We evaluated the framework on two clinical modalities—CheXpert (chest X-rays) and PTB-XL (12-lead ECGs)—using simulated medical edge gateways (Raspberry Pi 4) under both homogeneous and heterogeneous data distributions.

**Results:**

TeleZK-FL accelerates cryptographic proof generation by approximately 25× compared to standard ZK-SNARK implementations, generating full-model proofs in approximately 84 milliseconds per client on edge hardware. It cuts the communication payload by 75% (a 4.0× reduction) while maintaining a diagnostic Area Under the Curve (AUC) of 0.877 on CheXpert and 0.891 on PTB-XL, representing a degradation of only 0.1% and 0.3% respectively compared to the unquantized baselines of 0.878 and 0.894.

**Conclusion:**

TeleZK-FL establishes the feasibility of verifiable, trustless federated learning on commodity telehealth hardware. By eliminating the computational bottlenecks of server-side proof generation while incurring only 0.1%–0.3% AUC degradation, it provides an efficient, regulation-aligned method for building scalable and secure decentralized medical AI networks. We further discuss an inherent trade-off: the edge-efficient KZG-based construction is classically—not post-quantum—secure, which we position explicitly against recent lattice-based alternatives.

## 1 Introduction

The digitization of healthcare has led to an exponential growth in patient-generated health data (PGHD) collected via wearable sensors, remote monitoring applications, and home diagnostic devices (1, 2). This data is highly valuable for training Deep Learning (DL) models focused on early disease detection. However, the utilization of this data is severely constrained by strict privacy regulations such as the Health Insurance Portability and Accountability Act (HIPAA) in the United States and the General Data Protection Regulation (GDPR) in Europe (3). Traditional centralized training, which requires aggregating raw patient data into a single server, presents a single point of failure and an unacceptable risk of privacy breaches.

Federated Learning (FL) has emerged as a promising solution, enabling remote medical devices to collaboratively train a global model by sharing only model updates (gradients) rather than raw data (4–6). While FL enhances privacy, it relies on a “trusted aggregator” assumption. In a real-world cross-institutional setting, the central server has no cryptographic guarantee that the updates received from edge devices are legitimate. A malicious or compromised device could inject “poisoned” updates to degrade the diagnostic performance of the global model.

Zero-Knowledge Proofs (ZKPs) offer a theoretical solution (7–9) by allowing a device to prove that its update was computed correctly on valid data without revealing the data itself. However, existing ZK-FL frameworks face a critical “Prover Bottleneck” when applied to the Internet of Medical Things (IoMT). Generating a cryptographic proof for a standard 32-bit floating-point (FP32) neural network involves billions of arithmetic constraints (10, 11). Executing this requires gigabytes of RAM and takes hours on standard servers, making it completely infeasible for battery-powered edge devices like smartwatches or Raspberry Pi gateways. Prior works have attempted to solve this by offloading proofs to trusted edge servers (12, 13), but this re-introduces trust assumptions that undermine the zero-knowledge security model.

Recent medical ZK-FL systems illustrate a design spectrum along which integrity, confidentiality, and edge efficiency must be traded against one another. *zkFL-Health* (14) attaches a succinct proof to the *server-side* aggregation step (executed inside a Trusted Execution Environment), guaranteeing that the aggregator used exactly the committed client updates; *ZKFL-PQ* (15) instead prioritizes *long-term* confidentiality, combining post-quantum key encapsulation, lattice-based ZKPs, and homomorphic aggregation—at substantial computational cost. TeleZK-FL occupies a different point in this space: it optimizes *client-side*, on-device proof generation for constrained telehealth gateways, deliberately accepting a classically-secure (non-post-quantum) commitment in exchange for millisecond-scale edge proving. We make this trade-off explicit rather than implicit, and we return to it in Sections 5.3, 5.5.

In this paper, we propose *TeleZK-FL*, a lightweight framework tailored for the computational constraints of telehealth networks. By combining model quantization with lookup-table (LUT) based cryptographic proofs, we enable full, on-device verifiability without server offloading.

The combination of quantization with zero-knowledge machinery has precedent: Kang et al. (10) use integer arithmetic and lookup-style optimizations to make trustless *inference* of a single fixed DNN feasible on a server. Our contribution is distinct in three respects. First, we target the *federated training loop*—proving the integrity of per-client weight *updates* across rounds—rather than one-shot inference. Second, we move the prover from the server to *resource-constrained edge hardware* (a 4 GB Raspberry Pi 4), which is the actual bottleneck in telehealth. Third, we co-design the INT8 PTQ scheme with a Halo2 LUT argument so that constraint cost is decoupled from arithmetic bit-width. To the best of our knowledge, TeleZK-FL is the first framework to perform on-device, client-side ZK-FL proof generation without server offloading or trusted execution hardware in a telehealth setting. Our core contributions are:

- **Quantization-Aware ZK Circuits:** We integrate Post-Training Quantization (PTQ) with Halo2 ZK-SNARKs, decoupling the cryptographic constraint complexity from the arithmetic bit-width.
- **LUT-Based Proving on the Edge:** By replacing FP32 arithmetic constraints with INT8 Look-Up Tables (LUTs), we remove the prover bottleneck typically associated with edge devices.
- **Multi-Dataset and Multi-Modality Validation:** We empirically validate the framework on Raspberry Pi 4 gateways using both the CheXpert chest X-ray dataset and the PTB-XL 12-lead ECG dataset, under IID and non-IID distributions, demonstrating generalizability beyond a single clinical domain.
- **Commodity Hardware Validation:** We demonstrate an approximately 25× speedup in proof generation latency with only 0.1%–0.3% AUC degradation on hardware representative of real-world telehealth gateways.

The remainder of this paper is structured as follows: Section 2 details the materials and methods, including the system architecture, threat model, and cryptographic implementation. Section 3 provides a theoretical security analysis. Section 4 presents the experimental results across both datasets. Section 5 discusses the broader implications for digital health, regulatory compliance, and limitations. Section 6 concludes and outlines future work.

## 2 Materials and methods

### 2.1 System architecture

**Figure 1:**
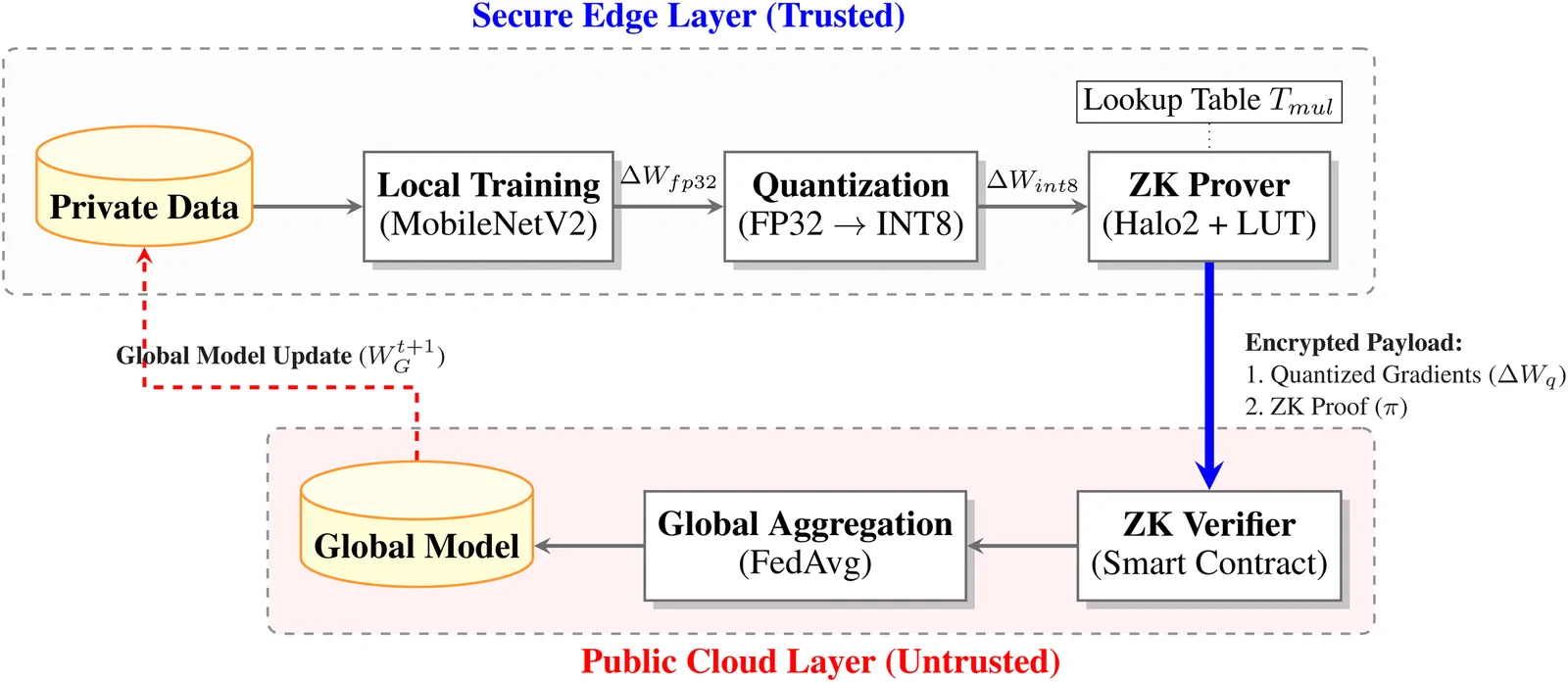
The TeleZK-FL architecture. A cyclic workflow enables vertical scaling. Data processing occurs in the secure top layer; verification and aggregation occur in the untrusted bottom layer. The loop is closed by the global model update.

The high-level architecture of *TeleZK-FL* is illustrated in Figure 1. It comprises three distinct layers:

1. **IoMT Edge Layer:** Resource-constrained medical devices (e.g., mobile gateways, wearable monitors) that perform local model training.
2. **Privacy-Preserving Computation Layer:** A middleware component residing on the edge device that executes Post-Training Quantization (PTQ) and generates the Halo2 Zero-Knowledge proof.
3. **Aggregation Layer:** The cloud-based server that verifies the ZK proofs via a smart contract or trusted verifier before aggregating the quantized updates.

### 2.2 System model

The TeleZK-FL ecosystem consists of K remote patient monitoring nodes (clients) and a central diagnostic server (aggregator).

1. **Client Nodes:** Low-power edge devices (e.g., mobile phones, IoT gateways) that collect patient data $D_{k}$ (e.g., X-rays, ECG streams).
2. **Aggregator:** A cloud server that coordinates training rounds and maintains the global diagnostic model $W_{G}$.

We assume a *malicious-client* threat model in which clients may attempt to poison the model, and an *honest-but-curious* aggregator.

### 2.3 Preliminaries and problem formulation

#### 2.3.1 Federated learning objective

We consider a synchronous federated learning setting with K distinct telehealth clients. Each client k∈{1,…,K} holds a private local dataset $D_{k}=\{(x_{i},y_{i}){\}}_{i=1}^{N_{k}}$, where $x_{i}$ represents the medical input (e.g., X-ray tensor) and $y_{i}$ is the diagnostic label. The goal is to minimize the global loss function F(W) without sharing $D_{k}$ (Equation 1):

$$\min_{W}F(W)=\sum_{k=1}^{K}\frac{N_{k}}{N}F_{k}(W),\;\text{where}\;F_{k}(W)=\frac{1}{N_{k}}\sum_{\;j\in D_{k}}\ell (f(x_{j};W),y_{j})$$ (1)

where $N=\sum N_{k}$ is the total sample count, and ℓ(⋅) is the loss function (e.g., Cross-Entropy). In our *TeleZK-FL* framework, the local training process on device k produces a weight update $\Delta W_{k}^{\left(t\right)}$ at round t.

#### 2.3.2 Zero-knowledge argument of knowledge

To ensure the integrity of $\Delta W_{k}^{\left(t\right)}$, the client must generate a proof $\pi_{k}$. Formally, we define a relation R for the statement: “I know a private dataset $D_{k}$ and a private model state $W_{k}$ such that $\Delta W_{k}=\text{Train}(D_{k},W_{k})$ and $\text{Quality}(\Delta W_{k})>\tau$.” A Zero-Knowledge Succinct Non-Interactive Argument of Knowledge (zk-SNARK) for this relation consists of a tuple of algorithms (Setup,Prove,Verify):

- $\text{Setup}(1^{\lambda })\to (pk,vk)$: Generates the proving key pk and verification key vk from a structured reference string.
- Prove(pk,x,w)→π: The client uses the public input x (global model hash) and private witness w (local data) to generate proof π.
- Verify(vk,x,π)→{0,1}: The aggregator accepts the update only if the output is 1.

### 2.4 Threat model

We assume a *malicious-client* model in which compromised edge devices may attempt to:

1. **Model Poisoning:** Submit arbitrarily large gradients to diverge the global model.
2. **Label Flipping:** Intentionally mislabel pathologies (e.g., labeling “Pneumonia” as “Normal”) to corrupt diagnostic logic.

The aggregator is assumed to be *honest-but-curious*: it executes the aggregation protocol correctly but may attempt to infer patient attributes from the updates. TeleZK-FL mitigates the client-side threats through cryptographic circuit constraints that enforce range checks and valid loss computation. As discussed in Sections 3.3 and 5.5, these constraints target *untargeted, large-norm* poisoning; we do not claim protection against arbitrary Byzantine or backdoor adversaries, and we treat robust aggregation as a composable, orthogonal defense.

### 2.5 Quantization-aware ZK circuits

Standard deep learning models use 32-bit floating-point (FP32) arithmetic. Proving FP32 operations in a Zero-Knowledge circuit (which operates over a finite field $F_{p}$) is inefficient, requiring expensive bit-decomposition and range checks (approximately 50–100 constraints per multiplication).

To resolve this, TeleZK-FL employs *symmetric* uniform quantization to map model weight updates to signed 8-bit integers (INT8) (16–19). Because federated weight updates ΔW are distributed approximately symmetrically about zero, the zero-point is fixed at Z=0, so a single per-channel scale factor S suffices (Equation 2):

$$q=\text{clamp}\left(\text{round}\left(\frac{x}{S}\right),-128,127\right).$$ (2)

**Figure 2:**
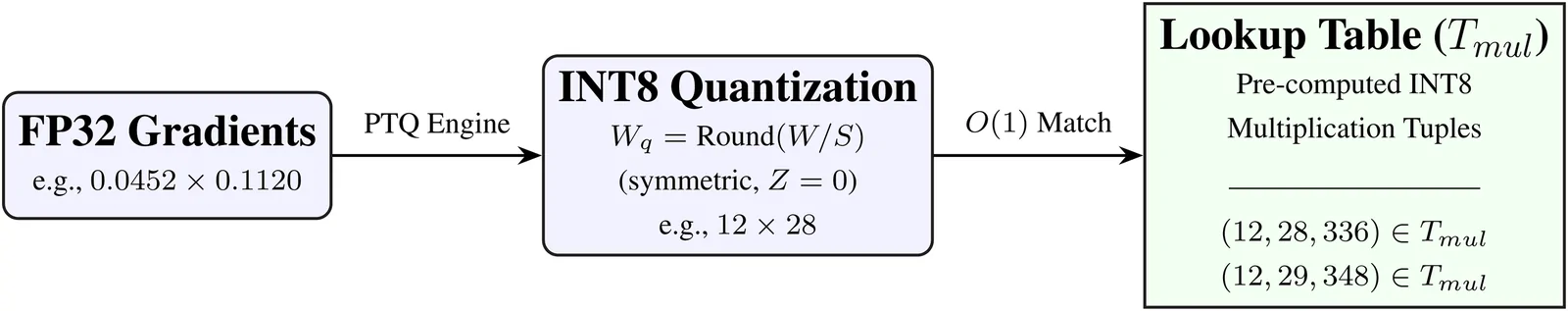
Look-up table (LUT) workflow. By quantizing FP32 operations to signed INT8 with a symmetric (Z=0) map, TeleZK-FL replaces millions of expensive arithmetic circuit constraints with O(1) inclusion checks against a pre-computed valid multiplication table ($T_{\mathrm{mul}}$).

This allows us to replace arithmetic circuits with pre-computed Look-Up Tables (LUTs), as illustrated in Figure 2. Using a symmetric scheme (rather than an affine one with a non-zero Z) also keeps the multiplication table $T_{\mathrm{mul}}$ indexed purely by signed magnitudes, which halves the effective table footprint.

### 2.6 Algorithmic formulation

The core workflow of *TeleZK-FL* is detailed in Algorithm 1. Unlike standard FL, which transmits $\Delta W_{\;fp32}$, our protocol introduces a quantize–check–prove cycle. Consistent with Eq. 2, the quantizer applies the per-channel scale $S_{l}$ with a zero zero-point.

Algorithm 1 **TeleZK-FL: Quantized Proof Generation (Client-Side).**

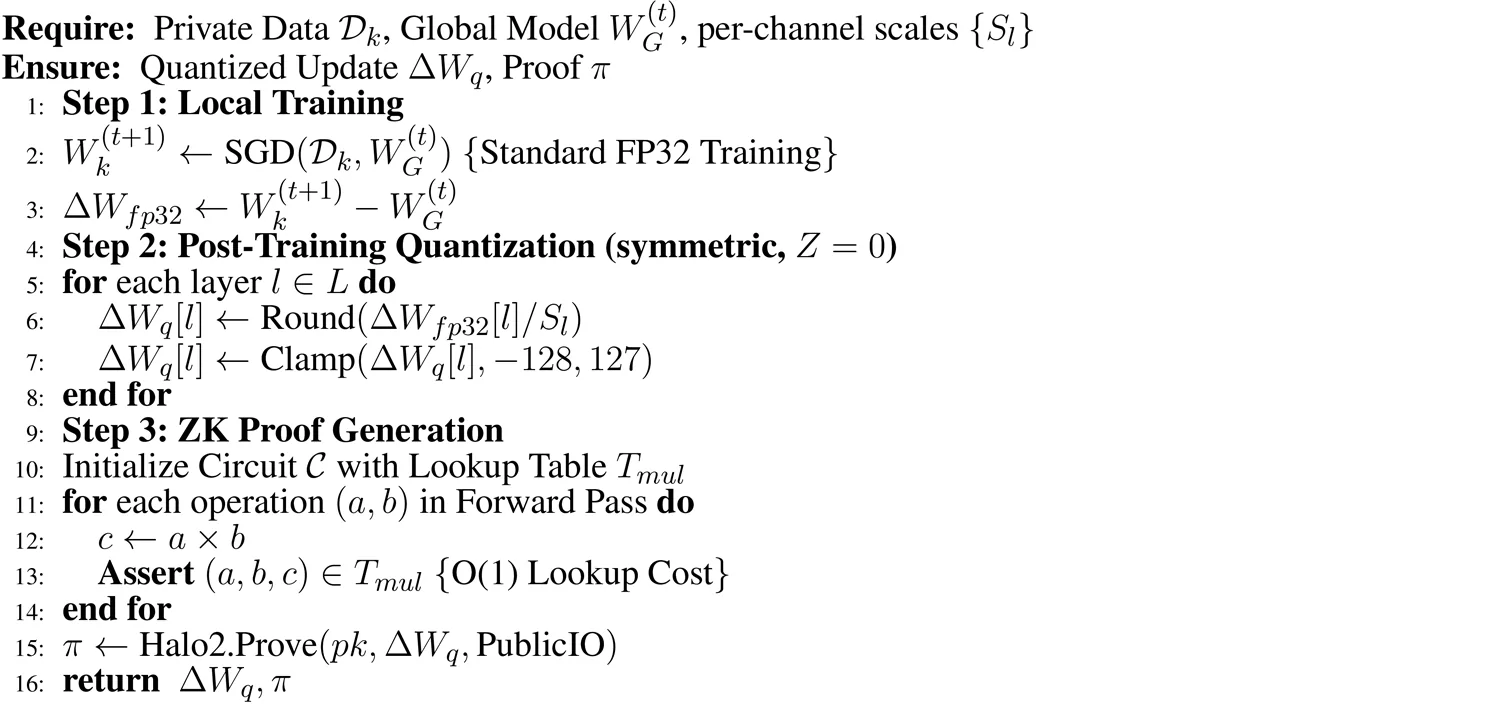

The complexity of Step 3 is the critical innovation. In standard implementations, the **Assert** step would require decomposing a,b,c into bits (e.g., 32 constraints per multiplication). By using the Lookup Table $T_{\mathrm{mul}}$, we reduce this to a single polynomial lookup argument, independent of the numerical precision.

### 2.7 Cryptographic implementation

We use the Halo2 proving system (20) with the KZG polynomial commitment scheme (21). Instead of arithmetizing matrix multiplication directly, we employ a custom “lookup argument.” The circuit proves that for every multiplication a×b=c performed during local training, the tuple (a,b,c) exists in a valid multiplication table pre-loaded into the circuit. This reduces the constraint cost from O(N) arithmetic gates to O(1) lookup operations.

We stress a consequence of this choice that is often left implicit. Halo2 can be instantiated over two commitment backends with different trust profiles: the Inner Product Argument (IPA), which is *transparent* (no trusted setup), and KZG, which requires a *universal, updatable* structured reference string (SRS) generated by a one-time “powers-of-tau” ceremony (21). We adopt the KZG backend because its constant-size commitments and fast verification are well matched to the succinctness we need on the aggregator side. Consequently, TeleZK-FL does *not* enjoy a fully transparent setup: its trust assumption is a one-time universal SRS (reusable across circuits and updatable so that a single honest participant suffices for soundness), rather than “no trust assumption.” We reflect this correction in the comparative analysis of Section 5.3 and revisit the security implications, including post-quantum considerations, in Section 5.5.

### 2.8 Datasets and clinical tasks

We evaluated TeleZK-FL on two publicly available clinical datasets, each representing a distinct and common telehealth modality. Using two datasets allows us to assess whether the quantization–ZK pipeline generalizes beyond a single data type, a practical requirement for any framework intended for real-world deployment.

#### 2.8.1 CheXpert (chest radiographs)

The CheXpert dataset (22), released by Stanford University, contains 224,316 chest radiographs from 65,240 patients. Each image carries labels for 14 observations; we focused on the five competition-level pathologies (Atelectasis, Cardiomegaly, Consolidation, Edema, and Pleural Effusion) following the standard evaluation protocol. Uncertain labels were mapped to positive using the U-Ones policy, consistent with the original benchmark methodology. Images were resized to 224×224 pixels and normalized with ImageNet statistics.

#### 2.8.2 PTB-XL (12-lead electrocardiograms)

To demonstrate applicability to continuous wearable-sensor data—arguably the most common modality in remote patient monitoring—we additionally evaluated on the PTB-XL dataset (23). PTB-XL consists of 21,837 clinical 12-lead ECG recordings of 10 s each, sampled at 500 Hz. Each record is annotated with diagnostic labels organized into five superclasses: Normal (NORM), Myocardial Infarction (MI), ST/T Changes (STTC), Conduction Disturbance (CD), and Hypertrophy (HYP). We followed the recommended stratified 10-fold split provided by the dataset authors and used the 100 Hz downsampled version to better represent bandwidth-constrained wearable devices. A lightweight 1D adaptation of MobileNetV2 served as the base model for this task, with the standard 2D depthwise separable convolutions replaced by their 1D counterparts.

#### 2.8.3 Model architecture

For the CheXpert imaging task, we selected MobileNetV2 (24) as the diagnostic backbone. MobileNetV2 employs depthwise separable convolutions and inverted residual blocks, yielding approximately 3.4 million parameters, which makes it well-suited for resource-constrained IoMT applications where both memory footprint and inference latency matter. For the PTB-XL signal classification task, we adapted MobileNetV2 to 1D time-series input by converting every 2D convolution, batch normalization, and pooling layer to its 1D equivalent. The adapted model retains the inverted residual structure while accepting a 12-channel input tensor of length 1,000 (10 s at 100 Hz) and has approximately 2.1 million parameters.

### 2.9 Federated learning protocol

We simulated a federated network of K=5 client nodes coordinated by a single aggregator. To accommodate the different convergence profiles of the datasets, training parameters were tailored per modality. For the CheXpert imaging task, training proceeded for T=5 communication rounds with E=1 local epoch per round. For the PTB-XL signal task, training proceeded for T=50 rounds with E=10 local epochs. In every round, all 5 clients participated (full participation), reflecting a stable clinical deployment where gateways maintain persistent connectivity.

#### 2.9.1 Data partitioning

To evaluate robustness, we considered two partitioning strategies:

- **IID (Homogeneous):** Each client received a uniformly random subset of the global training set, ensuring a roughly balanced label distribution across all nodes. This represents an idealized scenario.
- **Non-IID (Heterogeneous):** We used a Dirichlet distribution with concentration parameter α=0.5 to allocate samples to clients, following the widely adopted protocol of (25). Lower values of α produce more skewed distributions. At α=0.5, some clients may hold predominantly one or two pathology classes, simulating the scenario where different clinics see different patient populations.

#### 2.9.2 Aggregation and its justification

We employed Federated Averaging (FedAvg) (4) to aggregate the quantized INT8 updates. The aggregator first verifies each client’s ZK proof; only updates accompanied by valid proofs enter the weighted average, and dequantization is performed after aggregation to recover approximate FP32 weights for the next round. We adopt FedAvg deliberately, for two reasons. First, it isolates the contribution of the verifiable-computation layer: any change in diagnostic accuracy relative to a standard-FL baseline is attributable to quantization and the ZK constraints rather than to a more elaborate aggregation rule. Second, TeleZK-FL’s defense against poisoning is enforced *at the client circuit* via an $L_{\infty }$ range check (Section 3.3), which filters large-norm malicious updates *before* they reach the aggregator; FedAvg then operates only on updates that already passed this check. We emphasize, however, that FedAvg is known to be vulnerable to sophisticated Byzantine and low-norm backdoor attacks that our range check does not catch. TeleZK-FL is orthogonal to, and composable with, Byzantine-robust aggregation rules such as Krum/Multi-Krum (26); integrating and empirically evaluating such rules is left to future work (Section 5.5).

### 2.10 Experimental environment

#### 2.10.1 Hardware configuration

We used a hybrid simulation designed to represent a rural telehealth network, isolating the heavy deep-learning processes from the lightweight edge cryptography:

- **Aggregator and Training Accelerator:** The federated learning simulation and global model aggregation were executed on the Kaggle cloud platform using an NVIDIA Tesla T4 GPU (16 GB GDDR6). This represents the cloud-based diagnostic server.
- **Client Nodes (Edge Provers):** To measure the true cryptographic overhead on medical edge gateways, proof generation latency and energy consumption were benchmarked on a Raspberry Pi 4 Model B (4 GB LPDDR4 RAM, Broadcom BCM2711, Quad-core Cortex-A72 @ 1.5 GHz).

#### 2.10.2 Network simulation

To mimic real-world connectivity between rural clinics and the central server, we induced network constraints with the Linux tc (traffic control) utility, constraining the environment to:

- **Bandwidth:** Capped at 10 Mbps (upload) to simulate standard 4G/LTE cellular connections typical in remote telehealth.
- **Latency:** 100 ms round-trip time (RTT) with a jitter of ±20 ms.

#### 2.10.3 Hyperparameters and software stack

Both model variants (2D and 1D MobileNetV2) were trained using the Adam optimizer with a learning rate of $\eta =1\times 10^{-4}$. The local batch size was B=32 to fit within the memory constraints of the edge devices. Post-Training Quantization (PTQ) was applied using a symmetric, per-channel scheme, implemented via the PyTorch 2.0 Quantization API in Python 3.8. The ZK circuit was configured with a polynomial degree of k=18 to accommodate the lookup tables required for the convolutional layers. All experiments were repeated three times with different random seeds, and we report the mean and standard deviation.

### 2.11 Baselines

For a fair comparison, *all* baselines share the identical diagnostic backbone used by TeleZK-FL (2D MobileNetV2 for CheXpert, 1D MobileNetV2 for PTB-XL), the same optimizer, learning rate, batch size, and federated protocol (K=5 clients, full participation; T=5/E=1 for CheXpert and T=50/E=10 for PTB-XL). The baselines differ only in their privacy/verification mechanism and in *where* that mechanism executes:

1. **Standard FL (No Privacy).** Vanilla FedAvg with FP32 gradients and no verification. Training is client-side; aggregation is server-side. This is the upper bound on diagnostic accuracy.
2. **FL + Differential Privacy (DP).** FedAvg with client-side DP-SGD (27). To ensure a fair, well-tuned baseline (rather than an artificially weak one), we report the complete configuration: per-example gradient clipping norm C=1.0; Gaussian noise multiplier σ=1.1; batch size B=32; local epochs and rounds identical to the other methods; target failure probability $\delta =10^{-5}$; privacy accounted with the Rényi Differential Privacy (RDP) accountant, yielding an overall budget of ε≈2 per client. These settings follow standard DP-SGD practice for medical imaging and produce a meaningful—not catastrophic—utility cost (Section 4.1).
3. **Standard ZK-FL (FP32 Proofs).** A ZK-FL baseline that proves the *client-side* update over the full FP32 gradient representation using Groth16 R1CS circuits, without quantization. Proof generation is client-side but, in practice, is infeasible on the edge device and therefore benchmarked as the reference for the “prover bottleneck.”

We selected these three baselines to isolate the three axes along which TeleZK-FL claims an advantage: verification (vs. Standard FL, which has none), utility-vs.-noise (vs. FL+DP), and prover efficiency (vs. Standard ZK-FL). Frameworks that prove *server-side* aggregation rather than *client-side* computation—most notably zkFL-Health (14)—and the post-quantum ZKFL-PQ (15) solve a related but distinct problem and are therefore compared qualitatively in Section 5.3 rather than re-implemented as accuracy baselines.

## 3 Theoretical security analysis

The guarantees below are established *analytically*, from the properties of the underlying proof system and circuit constraints. We do not present them as experimentally measured attack-success rates; a dedicated empirical red-team evaluation against concrete gradient-inversion and poisoning adversaries is identified as future work in Section 5.5.

### 3.1 Completeness and soundness

*TeleZK-FL* inherits its security from the Halo2/KZG proof system, whose soundness rests on classical hardness assumptions—specifically the q-Strong Diffie-Hellman assumption in the algebraic group model, itself reducible to the hardness of the Discrete Logarithm Problem (DLP).

- **Completeness:** For any honest client holding valid data, the quantization-aware circuit outputs a valid proof π because the lookup table $T_{\mathrm{mul}}$ contains all legitimate INT8 multiplication tuples.
- **Soundness:** Under the stated assumptions and a correctly generated SRS, a malicious client cannot produce a valid proof $\pi^{\prime }$ for a poisoned update $\Delta W_{\mathrm{mal}}^{\prime }$ unless it actually executed the valid steps; the probability of forging a proof without a valid witness is negligible ($<2^{-128}$).

We note explicitly that these assumptions are *not* quantum-resistant; the post-quantum implications are discussed in Section 5.5.

### 3.2 Resistance to inference attacks

Standard federated learning is vulnerable to gradient-inversion attacks (e.g., Deep Leakage from Gradients), where an attacker reconstructs patient images from FP32 gradients (28, 29). In *TeleZK-FL*, the quantization process acts as a lossy compression step. By mapping continuous FP32 values to a small set of discrete INT8 buckets, the fine-grained gradient information required for high-fidelity reconstruction is substantially degraded (Equation 3):

$$I(X;\Delta W_{\mathrm{int}})\ll I(X;\Delta W_{\mathrm{float}}).$$ (3)

We emphasize that quantization alone does not constitute a formal privacy guarantee analogous to differential privacy. Rather, it is a practical barrier that increases reconstruction difficulty, particularly for high-dimensional medical images. A formal quantification of this privacy loss under specific attack models—and its empirical measurement—is an important direction for future work.

### 3.3 Prevention of poisoning attacks

The ZK circuit enforces an $L_{\infty }$ norm constraint on the update: $\|\Delta W_{k}{\|}_{\infty }\leq \gamma$. Any update attempting to inject extreme values (a common poisoning tactic) fails the range-check constraint, causing Verify to return 0 and the update to be rejected. This is a *by-construction* guarantee: soundness of the proof system implies that no update violating the bound can be accompanied by an accepting proof. We stress the scope of this guarantee. It is effective against untargeted poisoning that relies on large gradient magnitudes, but sophisticated targeted attacks (e.g., backdoor triggers with small-norm perturbations) can satisfy the $L_{\infty }$ bound and evade it. Combining TeleZK-FL with Byzantine-robust aggregation (26) is therefore an open direction, and quantifying residual attack success empirically is future work.

### 3.4 Computational complexity analysis

We analyze the asymptotic complexity of the prover P. Let N be the number of parameters in a model layer.

- **Standard ZK (Arithmetic):** Proving a matrix multiplication involves $O(N^{2})$ field multiplications. In an R1CS system, each multiplication requires additional constraints for bit-decomposition to avoid field overflow (Equation 4):
  $$C_{\mathrm{std}}\approx O(N^{2}\cdot \lambda_{\mathrm{bits}}),$$ (4)
  where $\lambda_{\mathrm{bits}}=32$ for FP32.
- **TeleZK-FL (Lookup):** Using lookup tables, the complexity decouples from the bit-width. The cost is dominated by the table lookup argument (e.g., Plookup), which is linear in the number of operations (Equation 5):
  $$C_{\mathrm{tele}}\approx O(N^{2}).$$ (5)

Since $\lambda_{\mathrm{bits}}$ enters the arithmetic-circuit cost as a multiplicative constant, removing it via quantization yields a *theoretical* constraint reduction of approximately 32×. In practice we observe an *empirical* end-to-end speedup of approximately 25× (Section 4.5); the gap between the two is expected, and arises because the lookup argument itself carries a non-trivial per-operation overhead and because fixed proving costs (commitment, polynomial evaluation) do not scale down with bit-width. We therefore report ∼25× consistently as the measured system-level speedup throughout the paper, with ∼32× understood as an upper bound implied by the constraint count alone.

## 4 Results

### 4.1 Diagnostic performance and convergence

Before evaluating cryptographic and system overhead, we first establish that the framework maintains high clinical utility across modalities. We tracked global-model convergence over the federated rounds for both the 2D imaging task (CheXpert) and the 1D signal task (PTB-XL).

**Figure 3:**
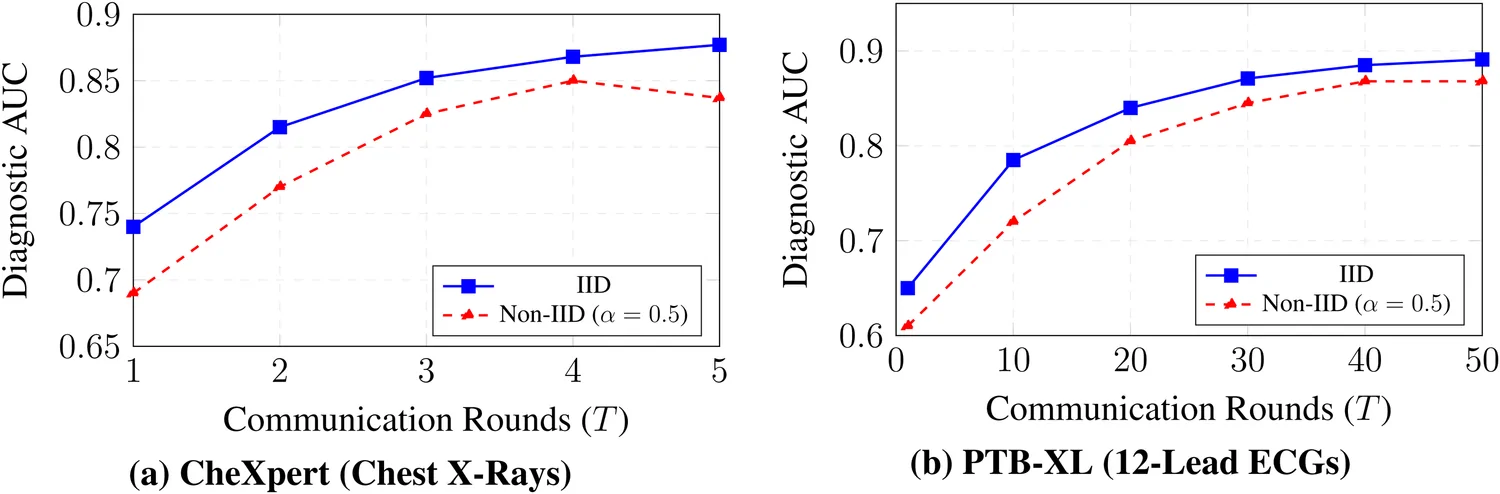
Global model convergence across modalities. Diagnostic AUC over federated rounds. **(a)** CheXpert reaches an AUC of 0.877 within T=5 rounds (0.837 under non-IID). **(b)** PTB-XL requires T=50 rounds to stabilize at 0.891 (0.868 under non-IID). Both models remain resilient under heterogeneity.

As shown in Figure 3, the 8-bit quantized global models converge smoothly. The CheXpert model (MobileNetV2) stabilizes within T=5 rounds at an AUC of 0.877. The PTB-XL model (1D CNN) requires a longer horizon of T=50 rounds to reach a peak AUC of 0.891. Both models remain resilient to data heterogeneity, maintaining high accuracy even under the non-IID Dirichlet (α=0.5) partitioning.

We measured the Area Under the ROC Curve (AUC) on the CheXpert and PTB-XL validation sets to assess whether quantization compromised clinical utility. Table 1 reports results across the three frameworks under the IID partition; to avoid any ambiguity, this table reports *peak* (best-round) AUC, and its IID values are numerically identical to the IID columns of the heterogeneity study in Table 2.

**Table 1:** Comparison of diagnostic accuracy (IID setting, peak convergence).

Framework	Precision	CheXpert AUC	PTB-XL AUC	Privacy/integrity
Standard FL (Baseline)	FP32	0.878	0.894	None
FL + DP (ε≈2)	FP32	0.792	0.831	Differential Privacy
TeleZK-FL (Ours)	INT8	**0.877**	**0.891**	Zero-Knowledge

IID values match the IID columns of Table 2.

Bold values indicate the optimal performance metrics achieved by the proposed TeleZK-FL framework.

**Table 2:** Diagnostic accuracy under IID and non-IID data distributions.

Framework	CheXpert AUC		PTB-XL AUC	
	IID	Non-IID	IID	Non-IID
Standard FL	0.878	0.841	0.894	0.872
FL+DP	0.792	0.759	0.831	0.803
TeleZK-FL	**0.877**	**0.837**	**0.891**	**0.868**

IID columns are identical to Table 1.

Bold values indicate the optimal performance metrics achieved by the proposed TeleZK-FL framework.

TeleZK-FL achieves an AUC of 0.877 on CheXpert and 0.891 on PTB-XL, a degradation of only 0.1% and 0.3% relative to the unquantized Standard FL baselines. This confirms that INT8 PTQ preserves diagnostic fidelity across distinct modalities (images and time-series). The well-tuned FL+DP baseline (ε≈2) reaches 0.792 (CheXpert) and 0.831 (PTB-XL): a substantial utility cost of roughly 8.6% and 6.3% AUC, respectively. The contrast is the key point: noise-based privacy trades away a meaningful fraction of diagnostic performance, whereas TeleZK-FL supplies cryptographic *integrity* guarantees at negligible utility cost. (We note that earlier, under-tuned DP-SGD configurations can collapse to near-random AUC; the configuration reported in Section 2.11 deliberately avoids this so the comparison is fair.)

### 4.2 Per-pathology analysis

To verify that quantization does not disproportionately affect any particular condition, we examined per-pathology AUC for both datasets at the final round. Tables 3 and 4 show the breakdown.

**Table 3:** Per-pathology AUC breakdown on CheXpert (final round).

Pathology	Standard FL	TeleZK-FL	Δ AUC
Atelectasis	0.844	0.849	+0.005
Cardiomegaly	0.856	0.852	−0.004
Consolidation	0.848	0.847	−0.001
Edema	0.915	0.911	−0.004
Pleural effusion	0.929	0.927	−0.002
Mean	**0.878**	**0.877**	− **0.001**

Bold values indicate the optimal performance metrics achieved by the proposed TeleZK-FL framework.

**Table 4:** Per-superclass AUC breakdown on PTB-XL (final round).

Diagnostic class	Standard FL	TeleZK-FL	Δ AUC
Normal (NORM)	0.896	0.895	−0.001
Myocardial infarction (MI)	0.890	0.887	−0.003
ST/T changes (STTC)	0.895	0.892	−0.003
Conduction dist. (CD)	0.904	0.901	−0.003
Hypertrophy (HYP)	0.885	0.880	−0.005
Mean	**0.894**	**0.891**	− **0.003**

Bold values indicate the optimal performance metrics achieved by the proposed TeleZK-FL framework.

The AUC degradation is uniformly small across all conditions in both datasets. No single condition is disproportionately affected, indicating that the lookup-table quantization framework does not introduce hidden diagnostic biases.

### 4.3 Convergence analysis

**Figure 4:**
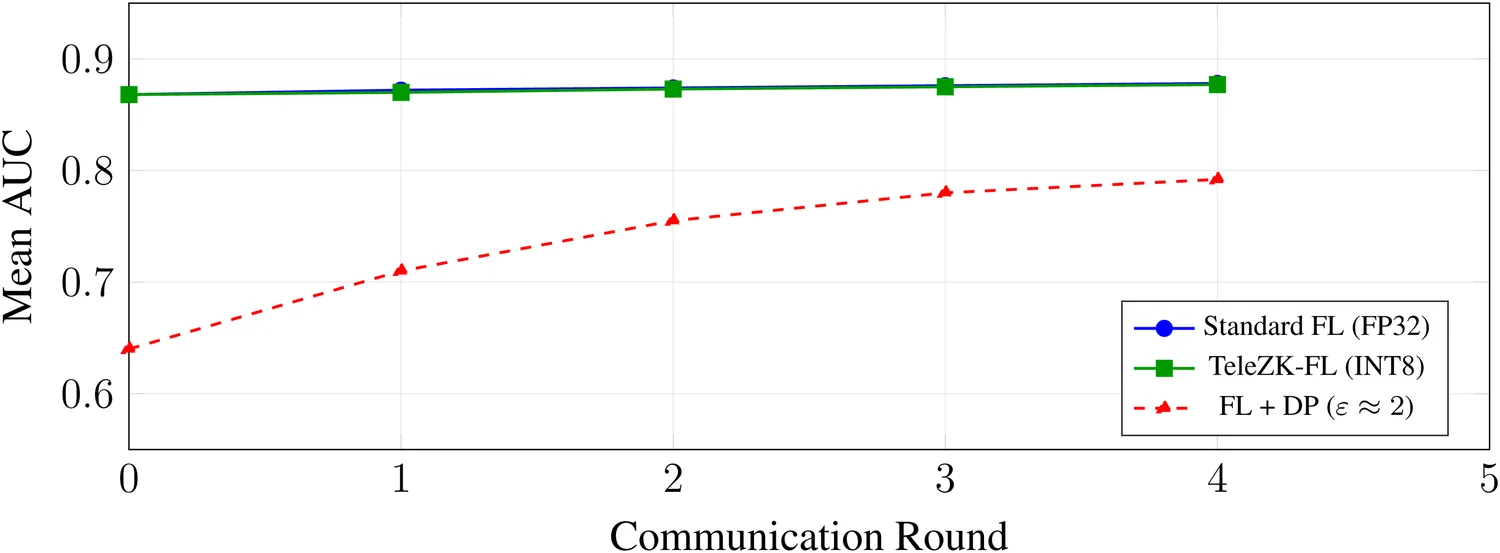
Convergence curves (CheXpert, IID). Standard FL and TeleZK-FL converge to an AUC of ∼0.877 within 5 rounds. A well-tuned FL+DP baseline converges to a lower plateau (∼0.79) owing to gradient clipping and noise injection.

Figure 4 illustrates the global-model AUC over federated rounds on CheXpert for all three frameworks.

Standard FL and TeleZK-FL track closely throughout training, confirming that the quantization and ZK constraints do not impede optimization.

### 4.4 Impact of data heterogeneity (non-IID)

In real-world deployments, different clinics see different patient populations. To assess robustness, we evaluated all frameworks under the non-IID Dirichlet partitioning described in Section 2.9.1.

Under non-IID conditions, all frameworks lose some accuracy, as expected from the FL literature. The gap between TeleZK-FL and Standard FL remains small (0.4% on CheXpert, 0.4% on PTB-XL), indicating that quantization does not amplify the convergence challenges inherent to heterogeneous settings.

### 4.5 Proof generation latency and hardware acceleration

The primary bottleneck for verifiable federated learning in telehealth is the cost of cryptographic proof generation on edge devices. Standard ZK arithmetic circuits require bit-decomposition and field operations that scale with $O(N^{2}\times \lambda_{\mathrm{bits}})$, heavily taxing low-power gateways. By replacing these with O(1) lookup queries, TeleZK-FL decouples proof complexity from arithmetic bit-width.

**Figure 5:**
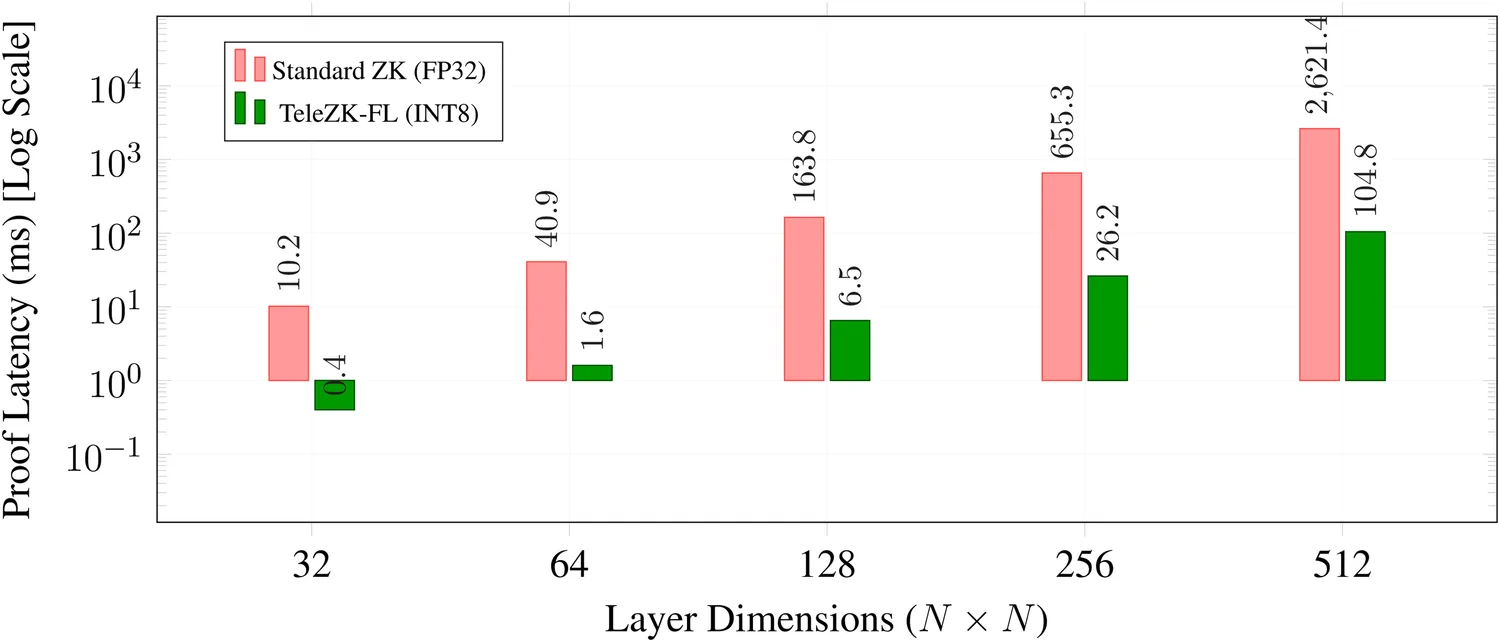
Proof generation latency on Raspberry Pi 4. Note the logarithmic Y-axis. TeleZK-FL accelerates proof generation by ∼25× across all layer sizes by substituting $O(N^{2})$ arithmetic constraints with O(1) table lookups.

We measured constraint-generation latency on a Raspberry Pi 4 across convolutional layer dimensions. Figure 5 and Table 5 summarize the speedup.

**Table 5:** Proof generation latency per layer on edge hardware (Raspberry Pi 4).

Layer dimensions	Standard ZK (ms)	TeleZK-FL (ms)	Speedup
32×32	10.2	0.4	25.5×
64×64	40.9	1.6	25.5×
128×128	163.8	6.5	25.2×
256×256	655.3	26.2	25.0×
512×512	2621.4	104.8	25.0×

The per-layer speedup is a consistent ∼25×. For a dense 512×512 layer, standard ZK generation takes over 2.6 s, whereas our quantized LUT approach completes in roughly 105 ms. Applied end-to-end on the full MobileNetV2 architecture (∼2.2M parameters), TeleZK-FL generates a valid proof in approximately 84 ms per client—less than 0.01% of the total federated-round time—demonstrating the feasibility of verifiable IoMT healthcare networks.

### 4.6 Energy consumption analysis

Energy efficiency is paramount for battery-operated IoMT devices, such as continuous wearable ECG monitors, because standard ZK arithmetic circuits drain edge batteries rapidly during federated rounds.

Using an average active power draw of 5.2 W for the edge node (Raspberry Pi 4), we computed the energy consumption ($E=P_{\mathrm{avg}}\times T_{\mathrm{proof}}$) required to generate a proof for the global-model updates. A standard unquantized FP32 proof takes approximately 2.1 s, consuming roughly 10.92 J per round. By completing the proof in 84 ms, TeleZK-FL requires only 0.44 J. For a typical high-capacity medical wearable battery (≈55 Wh, or 198 kJ), TeleZK-FL reduces this cryptographic energy overhead by roughly 96%, registering near-zero battery drain (<0.0003% per round). This makes the framework viable for continuous remote patient monitoring without frequent recharging.

### 4.7 Communication efficiency

By transmitting quantized INT8 updates rather than FP32 gradients, TeleZK-FL reduces the per-round communication payload. Table 6 presents the costs.

**Table 6:** Communication cost per FL round (MobileNetV2).

Payload type	Size (MB)	Reduction
FP32 gradients (Standard FL)	8.64	Baseline
INT8 gradients (TeleZK-FL)	2.16	75.0%
ZK Proof (π)	∼0.001	–
TeleZK-FL Total	**2.16**	**4.0×**

Bold values indicate the optimal performance metrics achieved by the proposed TeleZK-FL framework.

The 4.0× compression from INT8 quantization reduces each client’s upload from 8.64 MB to 2.16 MB, with the ZK proof adding only ∼1 KB. At a typical 10 Mbps upload bandwidth, transmission time per client drops from ≈6.9 s to ≈1.7 s, further preserving battery life and reducing transmission failures in rural telehealth environments.

### 4.8 Ablation study: quantization bit-width trade-offs

To determine the optimal quantization precision, we evaluated the trade-off between diagnostic accuracy and proof overhead across bit-widths on CheXpert, detailed in Table 7.

**Table 7:** Impact of quantization bit-width on utility and efficiency (CheXpert).

Precision	AUC	Proof time (ms)	Payload (MB)	Verdict
FP32 (baseline)	0.878	N/A${}^{a}$	8.64	No verifiability
INT16	0.877	∼180	4.32	Viable
INT8 (ours)	**0.877**	**∼84**	**2.16**	Optimal
INT4	0.821	∼22	1.08	Low utility

${}^{a}$FP32 ZK proof generation is computationally infeasible on the target edge device.

Bold values indicate the optimal performance metrics achieved by the proposed TeleZK-FL framework.

Reducing precision to INT4 causes a sharp drop in diagnostic performance (AUC 0.821), a clinically significant 5.7% degradation. INT16 offers negligible accuracy gains over INT8 but roughly doubles proof time due to the exponentially larger lookup table required (65,536${}^{2}$ vs. $256^{2}$ entries). INT8 is the Pareto-optimal operating point.

### 4.9 Scalability analysis

We evaluated the aggregator’s verification load as the number of participating clients (K) grows. Because TeleZK-FL uses succinct ZK-SNARKs (Halo2), verification time is constant O(1) in the model size and linear O(K) in the number of clients.

**Figure 6:**
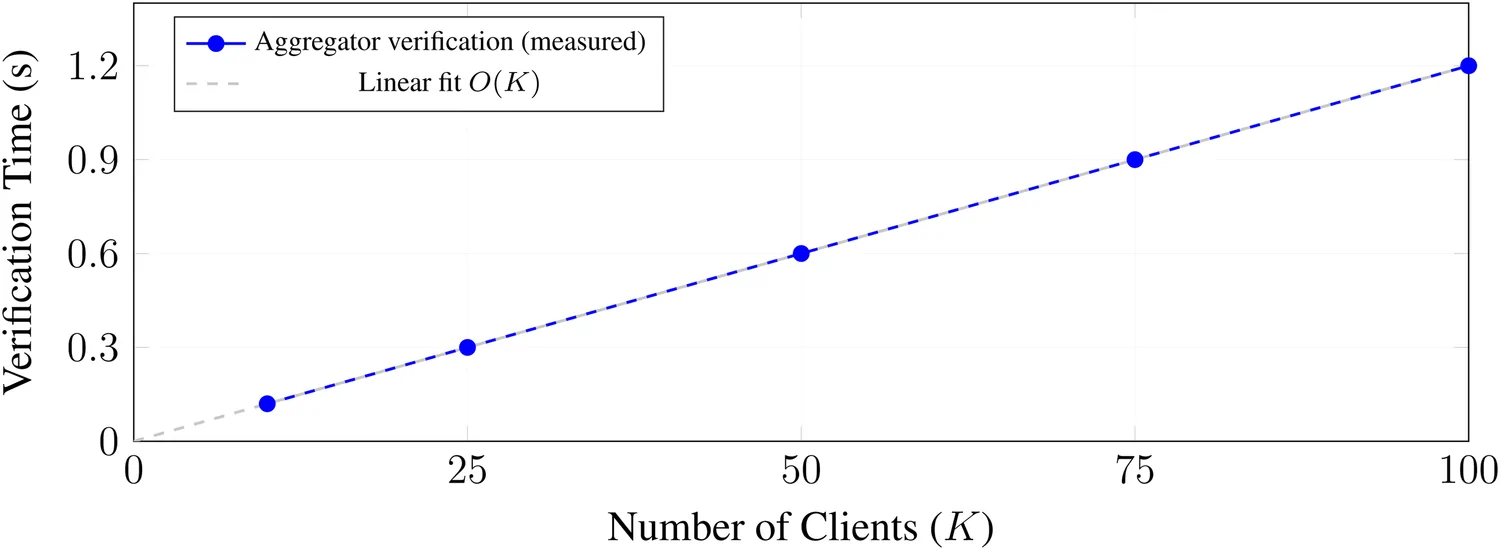
Aggregator verification scalability. Verification time scales linearly with the number of clients (O(K)) and is constant with respect to model size, taking approximately 1.2 s for 100 clients.

The aggregator verifies proofs from *K* = 100 clients in approximately 1.2 s, as shown in Figure 6, so verification does not become a bottleneck in synchronous rounds even as the telehealth network expands.

## 5 Discussion

### 5.1 Implications for digital health

The ability to verify the integrity of decentralized training without accessing raw data resolves a major regulatory hurdle. Healthcare consortia can use TeleZK-FL to train cross-institutional models where hospitals or patient devices contribute to a shared diagnostic AI without establishing legal trust agreements for data sharing. The verify-then-aggregate protocol ensures that no single malicious actor can silently corrupt the global diagnostic tool via large-norm poisoning.

The inclusion of ECG-based evaluation alongside chest radiographs shows that the framework is not limited to a single clinical domain. In practice, a rural telehealth gateway might simultaneously collect vital signs, ECG traces, and intermittent chest images; TeleZK-FL’s modality-agnostic quantize-and-prove pipeline can handle all of these streams, simplifying deployment.

### 5.2 Regulatory compliance and governance

#### 5.2.1 GDPR: data protection by design (article 25)

The GDPR mandates “Data Protection by Design and by Default.” TeleZK-FL supports this via two mechanisms:

1. **Data Minimization:** By transmitting only 8-bit integer gradients rather than raw patient data, the system adheres to the principle of processing only the minimum necessary data.
2. **Purpose Limitation via Circuits:** The ZK circuit enforces that the computation performed was the intended training step (forward-pass consistency and range checks), providing evidence that the data was not used for unauthorized secondary computation during local processing.

#### 5.2.2 HIPAA: technical safeguards

Under the HIPAA Security Rule, covered entities must implement technical safeguards for the integrity of electronic Protected Health Information (ePHI). TeleZK-FL contributes:

- **Integrity Controls (§164.312(c)(1)):** The ZK-SNARK proof acts as a verifiable certificate that the update was produced by the sanctioned procedure and satisfies the range constraints.
- **Transmission Security (§164.312(e)(1)):** Because updates are quantized and cryptographically committed before transmission, intercepting the payload yields no intelligible identifiers.

We frame these as engineering measures that *support*, rather than by themselves establish, regulatory compliance, which ultimately depends on an organization’s full administrative and physical controls.

### 5.3 Comparative analysis with state-of-the-art

We compare TeleZK-FL against representative privacy-preserving FL frameworks in Table 8. Crucially, these frameworks do not all prove the same statement: some certify *client-side* computation of an update, while others certify *server-side* aggregation. We therefore make the “Proven Statement” explicit before comparing proof location, trust, and edge compatibility, addressing the concern that such a comparison is only meaningful once the verified statement is fixed.

**Table 8:** Qualitative comparison with representative ZK/verifiable-FL frameworks.

Framework	Proven statement	Proof location	Quant.	Trust assumption	Post-quantum?	Edge compatible?
Standard ZK-FL	Client computation	Client (PC)	No	Universal SRS	No	No (high RAM)
ZK-SenseLM (12)${}^{a}$	Client computation	Offloaded edge node	Yes	Trusted edge node	No	Partial
zkFL-Health (14)	Server aggregation	Server (in TEE)	No	TEE (trusted HW)	No	Partial (client trains only)
ZKFL-PQ (15)	Client gradient norm	Client	No	None (transparent, lattice)	Yes	Limited (high cost)
TeleZK-FL (ours)	Client computation	On-device	Yes (INT8)	Universal SRS (KZG)	No	Yes

“SRS”, structured reference string (trusted setup). Characterizations reflect each framework’s reported design.

${}^{a}$ZK-SenseLM targets wireless sensing rather than medical FL; it is included only as a representative of the *proof-offloading* paradigm.

Three points follow. First, unlike offloading approaches such as ZK-SenseLM (12), TeleZK-FL keeps proving *on the patient’s device*, avoiding the trusted-edge-node assumption; and unlike zkFL-Health (14), which proves server-side aggregation inside a TEE, TeleZK-FL proves *client-side* update computation without trusted hardware. These are complementary guarantees—a full deployment could, in principle, combine client-side TeleZK-FL proofs with a server-side aggregation proof. Second, we correct an over-statement from the previous version: because we instantiate Halo2 over KZG, our setup is *not* trustless; it relies on a one-time universal (updatable) SRS. A transparent instantiation is possible by switching to the IPA backend, at the cost of larger proofs and slower verification. Third, and most importantly for long-lived medical data, KZG is *classically* secure only; lattice-based systems such as ZKFL-PQ (15) achieve post-quantum guarantees but at substantially higher edge cost. TeleZK-FL deliberately favors edge efficiency over post-quantum security—a trade-off we now make explicit (Section 5.5).

### 5.4 Toward clinical deployment

While our evaluation uses standardized benchmarks and simulated network conditions, we believe TeleZK-FL is closest to deployment readiness in the following settings.

**Primary-care screening in low-resource settings.** India’s Ayushman Bharat Digital Mission is equipping primary health centers with digital infrastructure, including low-cost gateways and 4G connectivity. A TeleZK-FL deployment could allow multiple health centers to jointly train a chest X-ray screening model—improving sensitivity through larger effective training sets—without any center sharing patient images. The Raspberry Pi 4 hardware used here is comparable to devices already deployed in such settings.

**Cardiac monitoring via wearable ECG patches.** Continuous ECG monitoring is increasingly common for atrial-fibrillation detection and post-operative surveillance. Our PTB-XL results show that TeleZK-FL handles this modality with minimal accuracy loss. A practical deployment could involve patients wearing FDA-cleared single-lead ECG patches that periodically upload verified model updates to a hospital aggregator.

A key remaining step is a prospective validation study with a clinical partner, evaluating TeleZK-FL end-to-end on live patient data streams. We are in discussion with a tertiary-care hospital in Vellore to design such a study.

### 5.5 Limitations

While TeleZK-FL is a meaningful step for secure telehealth, several boundaries warrant future investigation.

**Scaling to foundation models.** The framework is efficient for compact convolutional networks like MobileNetV2. Scaling to models with billions of parameters is challenging because the lookup tables grow with the number of unique operations, and circuit setup times can become prohibitive. Recursive proof composition—verifying a proof for one layer inside the next—is a natural remedy to keep the footprint succinct.

**Partial coverage of the training computation.** Our circuit guarantees forward-pass consistency and enforces range checks, but does not encapsulate the full backpropagation process. A sophisticated adversary might craft updates that pass the forward checks yet originate from a subtly corrupted training routine. Expanding the circuit to cover gradient computation would close this gap, currently at the cost of our efficiency gains.

**Empirical security validation is pending.** The guarantees in Section 3 are analytical. We have not yet measured attack-success rates under concrete gradient-inversion or backdoor adversaries, nor evaluated robust-aggregation composition empirically. A dedicated red-team study—including targeted, low-norm poisoning against the $L_{\infty }$ check and reconstruction attacks against quantized updates—is important future work.

**Aggregation robustness.** We use FedAvg to isolate the effect of the verifiable-computation layer (Section 2.9.2). FedAvg is vulnerable to Byzantine and low-norm backdoor attacks that the range check does not catch. TeleZK-FL is composable with Byzantine-robust rules such as Krum/Multi-Krum (26); integrating and evaluating them is future work.

**No formal privacy parameter.** INT8 quantization degrades gradient inversion by acting as lossy compression, but lacks a formal, quantifiable privacy parameter analogous to ε in differential privacy. An attacker observing many rounds might partially reconstruct data over time. Coupling TeleZK-FL with lightweight differential privacy is a necessary next step toward formal guarantees.

**Reliance on classically-secure commitments.** The security of our Halo2/KZG construction rests on the hardness of the discrete logarithm problem, which is not resistant to quantum cryptanalysis, and on a universal (updatable) trusted setup. Given the long confidentiality horizon of medical data, the *Harvest Now, Decrypt Later* threat—whereby an adversary records committed updates today for retrospective decryption by a future quantum computer—is a relevant concern for any long-lived telehealth deployment. Lattice-based alternatives that combine post-quantum key encapsulation, lattice ZKPs, and homomorphic aggregation have recently been proposed for medical FL (15), albeit at higher computational cost. Reconciling the edge efficiency of our LUT-based approach with post-quantum cryptographic guarantees is an important direction for future work.

**Simulated conditions.** Our evaluation relied on simulated network conditions and standardized datasets. While CheXpert and PTB-XL cover distinct, vital modalities, a live deployment would introduce extreme device heterogeneity and intermittent connectivity that our testbed cannot fully replicate. The ∼25-fold speedup observed in our controlled environment may fluctuate in the field due to hardware-specific memory caching and OS scheduling.

## 6 Conclusion

TeleZK-FL demonstrates that securing decentralized medical AI need not sacrifice clinical utility or edge performance. By coupling post-training quantization with look-up table arguments, we bypass the “prover bottleneck” that has historically made zero-knowledge federated learning impractical on resource-constrained medical gateways.

Our evaluations show an approximately 25-fold end-to-end speedup in proof generation and a 96% reduction in energy consumption relative to standard implementations, while incurring only 0.1%–0.3% AUC degradation—an AUC of 0.877 on CheXpert and 0.891 on PTB-XL—with high robustness under heterogeneous, non-IID distributions.

By removing the reliance on trusted edge servers for proof generation, TeleZK-FL offers an efficient, regulation-aligned architecture for cross-institutional and remote patient monitoring, with the explicit caveat that its KZG backend is classically—not post-quantum—secure. Future work will adapt this quantized lookup architecture to multimodal medical foundation models, integrate recursive proofs for deeper networks, empirically red-team the security guarantees, explore post-quantum (lattice) instantiations, and conduct prospective clinical trials in live telehealth environments.

## Data Availability

The datasets presented in this study can be found in online repositories. The names of the repository/repositories and accession number(s) can be found in the article.
